# Prevalence of* Chlamydia trachomatis* among Childbearing Age Women in India: A Systematic Review

**DOI:** 10.1155/2016/8561645

**Published:** 2016-09-08

**Authors:** Kalpana Betha, Jamie M. Robertson, Gong Tang, Catherine L. Haggerty

**Affiliations:** ^1^SHARE India, MIMS, Hyderabad, India; ^2^STRATUS Center for Medical Simulation, Brigham and Women's Hospital, Boston, MA, USA; ^3^Department of Biostatistics, Graduate School of Public Health, University of Pittsburgh, Pittsburgh, PA, USA; ^4^Department of Epidemiology, Graduate School of Public Health, University of Pittsburgh, Pittsburgh, PA, USA

## Abstract

*Background*. Infection with* Chlamydia trachomatis* (CT) can lead to reproductive sequelae. Information on the general population of childbearing age women in India is sparse. We reviewed the literature on CT prevalence within the general population of reproductive aged women in order to improve the efforts of public health screening programs and interventions.* Objective*. To conduct a literature review to determine the prevalence of* Chlamydia trachomatis* among childbearing age women in India.* Search Strategy*. Ovid Medline and PubMed databases were searched for articles from January 1, 2003, through December 31, 2014. Search terms included “*Chlamydia trachomatis*”, “CT”, “prevalence”, “India”, and “sexually transmitted infections”.* Selection Criteria*. Studies on prevalence data for CT among women of childbearing age (15–45) living in India were included.* Data Collection and Analysis*. Articles that met the inclusion criteria were extracted by two readers and discrepancies solved through discussion.* Results*. Reported prevalence of active CT infection among lower risk groups ranged from 0.1% to 1.1% and in higher risk group from 2.7% to 28.5%.* Conclusion.* CT prevalence among women in India is comparable to other countries. Screening programs to prevent adverse outcomes among Indian women of childbearing age and their offspring are warranted.

## 1. Background

Worldwide,* Chlamydia trachomatis* (CT) is the most common bacterial sexually transmitted infection (STI), with approximately 105.7 million new infections occurring annually [1]. Untreated CT can lead to serious reproductive sequelae for women, including pelvic inflammatory disease (PID) [2, 3], tubal factor infertility [1, 3], ectopic pregnancy [4, 5], and an increased risk of acquiring other STIs [6]. In addition, pregnant women are at an increased risk of having a preterm birth and giving birth to a low birth weight infant [7, 8]. Neonates born to infected mothers are more likely to have pneumonia and neonatal conjunctivitis [8].

As the rate of CT around the world has been climbing over the last decade [1], public health agencies have increasingly been concerned with identifying and screening populations at high risk and those who have the most serious consequences from untreated infections. Recommendations for screening have focused largely on adolescent and pregnant women [9, 10] due to the increased risk of long term adverse consequences of unidentified infections. These programs are based on information about the population based prevalence of CT and distribution of infection among age groups, ethnicities, and other subgroups of interest. Few studies have been conducted to determine the effectiveness of screening programs to reduce sequelae and have shown mixed results, but some have shown a decrease in PID, infertility, and ectopic pregnancy [11, 12].

In India, no national data source contains information about the prevalence of CT. Though many studies of STIs have been done throughout the country, most of this piece of information has focused on high risk populations, mainly HIV positive women and female sex workers [13, 14]. Information on the general population of childbearing age women is sparse and spread between studies conducted in diverse geographical settings. We sought to systematically review the existing literature on CT prevalence within the general population of reproductive aged women in India in order to improve the efforts of public health screening programs and interventions.

## 2. Methods

This review was performed in accordance with the Preferred Reporting Items for Systematic Reviews and Meta-Analyses (PRISMA) guidelines [21].

### 2.1. Selection Criteria

Studies were included in our review if they were published between January 1, 2003, and December 31, 2013. Date restrictions were used to ensure that studies were comparable in terms of available diagnostic technology, cultural norms during the study period, and overall population living in India and centered on women of childbearing age (15–40 years). Studies were excluded if they focused exclusively on sex workers, HIV positive women, or men. Studies of both men and women were included if data were stratified by sex.

### 2.2. Search Strategy

We searched PubMed and Ovid Medline for articles pertaining to CT prevalence in India. Search terms included “*Chlamydia trachomatis*”, “CT”, “prevalence”, “India”, and “sexually transmitted infections”. Abstracts were assessed and the full texts of articles that might meet the inclusion criteria were obtained. Two reviewers (Kalpana Betha and Jamie M. Robertson) read and determined the eligibility of each article. A third reviewer (Catherine L. Haggerty) was available to solve any disagreements. Reference lists of relevant articles were cross-referenced to identify additional studies.

### 2.3. Data Extraction

Information from articles that met the inclusion criteria was extracted by two readers (Kalpana Betha and Jamie M. Robertson) and compared for agreement. Any discrepancies were solved through discussion. Information extracted included the following: author(s), journal, year of publication, study design, participant demographics, sample size, setting, CT testing method, and prevalence findings.

## 3. Results

### 3.1. Search Results

**Figure 1:**
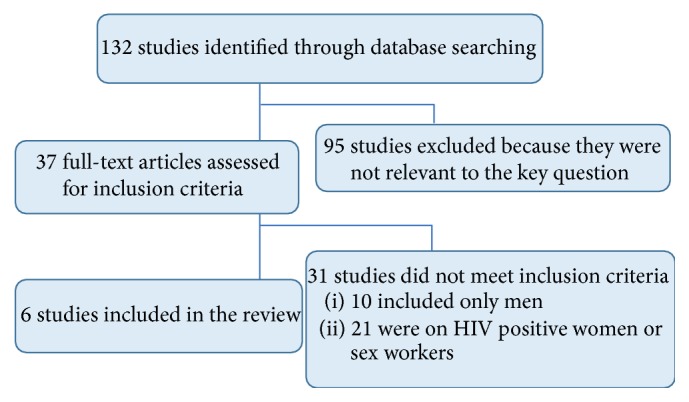


Our database search yielded a total of 132 studies (see Figure 1). Of the 38 deemed to be relevant to the key question, 21 were on HIV positive women or sex workers and 10 included only male subjects. The six remaining studies were included in this analysis (Table 1) [15–20].

### 3.2. Population Characteristics

Three studies [16, 17, 19] utilized patients seen in outpatient gynecology clinics in hospitals and one [18] from a combined obstetrics and gynecology outpatient hospital clinic. The following recruitment sites were utilized each in one study: community [20] and antenatal care clinic [1]. Three studies were conducted in North India [16, 18, 19], with two studies done in Delhi [16, 19] and one in Bhubaneswar, Orissa [18]. The remaining three studies were conducted in South India [15, 17, 20]. Two were conducted in the state of Tamil Nadu, one in the city of Vellore [15], and one in the districts of Tanjore, Ramnad, and Dindigul [20] and used the “probability proportion to size” cluster survey method in these districts. The final study was conducted in northern Karnataka [17]. One study recruited women <18 years of age [20] and all the remaining recruited adult women [15–18]. Mean or median age was reported in four of the studies and varied from 25.8 to 31.5 years [15–18].

### 3.3. Diagnostic Method

All 6 studies employed polymerase chain reaction (PCR) testing as a method of CT diagnosis [15–19]. One study combined multiple methods of diagnostic testing, including a combination of PCR and enzyme-linked immunosorbent assay (ELISA) [20].

### 3.4. Reported Prevalence of CT

The prevalence of active chlamydial infection assessed by PCR or NAAT was greatest among populations considered to be at a generally higher risk for sexually transmitted infection, including symptomatic and asymptomatic women presenting to obstetrics and gynecology, gynecology, or STI clinics. In four studies, the rate of CT ranged from 2.7% to 23% [16–19] with variation largely explained by age and the highest rates reported among younger women. Of note, the highest rate (28.5%) was documented among 18–25-year-old patients at a hospital gynecology clinic in New Delhi [19]. In this study of 280 symptomatic and asymptomatic women, rates were considerably lower among 25–35-year olds (7.6%) and 35–45-year olds (3.2%). In a study of 108 married symptomatic women with a mean age of 32 years in Orissa, the prevalence rate reported was 7.0% [18]. A study of 412 symptomatic women with a mean age of 31 years attending gynecology clinics in Karnataka State demonstrated a prevalence of 2.7% [17]. Among 335 symptomatic, nonpregnant women aged 18–60 attending a gynecology clinic in Delhi, 23% tested positive by PCR. The vast majority of positive cases (>92%) were of reproductive age 18–41 years [16]. The lowest prevalence of active chlamydial infection was reported among groups of women considered at a generally low risk for sexually transmitted infection, including a population based sample of 1,066 symptomatic and asymptomatic pregnant women in Tamil Nadu (1.1%) [20].

## 4. Discussion

This is the first systematic review of CT prevalence among childbearing age women in India which does not focus on women who are not part of a high risk group, such as those who are HIV positive or are sex workers. We found the reported prevalence of active CT infection detected using molecular techniques among lower risk groups including pregnant women presenting for antenatal care and a general population based study to be lowest, ranging from 0.1% (95% CI 0–0.38%) to 1.1% (95% CI 0.5%–1.7%). Prevalence of current infection among populations considered to be of higher risk including symptomatic and asymptomatic women presenting to obstetrics and gynecology, gynecology, or sexually transmitted disease clinics was higher, ranging from 2.7% to 28.5%. Variation in this higher risk group was largely explained by age, with the highest rates among younger women.

Though we did not include studies of known high risk populations, the majority of studies included in this review were done among women presenting to either gynecologic or STI clinics, the majority of whom reported symptoms of sexually transmitted and reproductive tract infections. This may bias our review toward reporting a higher prevalence of CT. However, studies including symptomatic and asymptomatic women taken from other clinic settings, such as antenatal clinics, and population based studies mostly found results similar to what has been seen in population based studies in the United States and other countries. In the United States, the prevalence of CT among women aged 14–39 years is 2.2% (95% CI: 1.4–3.4%) [22], with higher rates reported among subgroups [20, 22, 23]. Based on CDC estimates from national surveys conducted from 1999 to 2008, the overall prevalence was 6–8% among sexually active females aged 14–19 years. The highest age specific rates of reported Chlamydia infection in 2010 were among those aged 15–19 years (3,378.2 cases per 100,000 females) and 20–24 years (3407.9 cases per 100,000 females). Two studies estimated that only about 30% of women with laboratory confirmed* Chlamydia* infection develop symptoms [24, 25]. And given the relatively slow replication cycle of the organism, symptoms may not appear until several weeks after exposure in those persons who develop symptoms. Thus, asymptomatic infection is common, and rates in India are similar to if not higher than those in the US.

The current practice in India is to conduct opportunistic screening among symptomatic women. As asymptomatic infection is common, it would be optimal to offer screening for both symptomatic and asymptomatic women, including pregnant women who present to antenatal clinics, in order to provide treatment, prevent sequelae, and decrease the chances of CT being spread throughout the community. Additionally, it would be optimal to test, treat, and counsel partners. If partners are unwilling or unable to access medical services, expedited partner therapy should be advocated and women should be instructed to abstain from sexual intercourse for 7 days until partners have completed treatment. The current practice for partner management in India is client-initiated partner notification [26]. In a study by Pratibha et al. [27], contact tracing and treatment remained a major problem and less than one-third of women showed a positive response to obtain blood samples from their partners. A high prevalence of infection (40%) was found among partners, suggesting that sexual partners should be actively screened and treated to prevent reinfection rates. Stigma management strategies should be applied like selective disclosure and selective encouragement of others to test to avoid stigmatizing reactions.

We found that the majority of published information related to the prevalence of CT among women comes from obstetrics and gynecologic clinics. Most symptomatic women in India present to such facilities instead of STI specific clinics [17] making these an ideal location to screen and diagnose the greatest number of women with little monetary or resource investment. However, this setup does not allow for screening of asymptomatic women, as they do not present for regular visits. Given that the majority of women with CT are asymptomatic [28], this method of identification and testing is insufficient to prevent adverse outcomes related to untreated CT infections. Efforts to screen asymptomatic women in the community, in both rural and urban settings, are necessary to prevent transmission and sequelae. Community-based trials of available screening techniques, including education counseling, testing options, and treatment, are needed to identify programs that are culturally acceptable and work within the existing health care infrastructure.

This focus is especially important given the changing cultural climate in India. Increasingly, adolescents, especially males, are engaging in premarital sexual activity [29, 30]. A lack of knowledge about condoms and other contraceptive methods combined with a growing number of men who engage in first-time sexual encounters with sex workers creates an environment where the rate of STIs will likely continue to rise over the next decade [29, 30]. As the majority of studies included in this review did not provide rates stratified by age, it is impossible to say whether the prevalence of CT is already higher among the younger generations. Only one study that did it found individuals in the age groups of 18–25 [19] years having the highest reported rates among their respective populations, which is consistent with data from other countries [23, 31, 32]. As CT causes permanent damage to fallopian tubes, untreated CT infection in adolescents could have long-term consequences which may not manifest themselves until women later try to conceive. Increased efforts to screen and treat this younger population are thus important in order to preserve fertility.

Increased screening efforts can prevent a variety of sequelae associated with CT. In the United States, the Centers for Disease Control and Prevention (CDC) recommends annual CT screening for all women aged 25 years and younger. In addition, it recommends screening all pregnant women at the first prenatal visit and second screening during the third trimester for women ≤25 years of age and those identified to be of high risk [10]. Other countries, including Australia [9, 33], the United Kingdom [34], and Canada [24], have developed similar guidelines. Similar testing guidelines would be difficult to implement in India due to lack of providers in many rural areas, an already overburdened system, and lack of interaction between healthy young people and providers. However, antenatal visits offer an important point of contact that could be utilized for counseling and testing. Such a program could prevent adverse outcomes related to pregnancy, such as preterm birth, and help prevent neonatal outcomes such as neonatal conjunctivitis and pneumonia. The relatively small number of studies in the general population on CT prevalence highlights the need for additional studies on CT prevalence and incidence in order to guide screening and intervention trials and programs.

Finally, in order to better understand the true burden of CT on the population, more community-based studies of asymptomatic individuals are needed. In addition, identification of risk factors unique to populations in India may help identify targets for ongoing community-based studies that would provide an important way to identify CT in nonpregnant populations and prevent additional infections.

## Figures and Tables

**Table 1:** Characteristics of the studies addressing *Chlamydia trachomatis* (CT) prevalence among childbearing age women in India.

Author	Year	Setting	Location	Population	*N*	Age	Diagnostic method	Sample source	Prevalence (95% CI)^1^	Validity
Vidwan et al. [15]	2012	ANC clinic	Tamil Nadu	Pregnant (≥28 weeks' gestation)	784	Mean: 25.8 Range: 18–39	NAA	Endocervical	0.1% (0–0.38%)	This is the largest study on CT prevalence among healthy pregnant mothers in South India. But project sample population may not represent local delivering female population.

Patel et al. [16]	2010	GYN clinic	New Delhi	Symptomatic, nonpregnant	335	Median: 29 Range: 18–60	PCR	Endocervical	23.0%	Used in-house PCR method which was cost-effective; included only symptomatic patients.

Becker et al. [17]	2010	GYN clinics	Karnataka	Symptomatic	335	Mean: 30.7	PCR	Endocervical	2.7%	It has good sample size;women were recruited from gynecology clinics; itmay lead to enrollment of low risk women.

Dwibedi et al. [18]	2009	OB/GYN clinic	Orissa	Symptomatic	71	Mean: 31.5 (SD: 7.3)	PCR	Endocervical	7.0%	It has small sample size. It is the first report from the region. It may help clinicians of the region in treating cases with similar symptoms.

Singh et al. [19]	2003	GYN clinic	New Delhi	Symptomatic and asymptomatic	280	18–25 25–35 35–45^2^	PCR	Endocervical	28.5% 7.6% 3.2%	Studied age-wise prevalence rate and determined most prevalent serovars of CT.

Joyce et al. [20]	2004	Community^3^	Tamil Nadu	Symptomatic and asymptomatic	1066	Range: 15–45	PCR ELISA	Urine Blood	1.1% (0.5–1.7%) 3.3% (1.9–4.7%)	It is the first population based study in India. It has the largest sample size. It used two methods to determine prevalence.

^1^95% CI reported when available from paper.

^2^Reported only as age groups; no measure of central tendency available.

95% CI: 95% confidence interval; ANC: antenatal care; OB/GYN: obstetrics and gynecology; GYN: gynecology; SD: standard deviation; NAA: nucleic acid amplification; PCR: polymerase chain reaction; ELISA: enzyme-linked immunosorbent assay; DFA: direct fluorescent antibody; STI: sexually transmitted infection.

^3^Representative sample taken from 3 randomly selected districts by using the probable proportional to size cluster survey method.
